# Evaluation of bone marrow aspirates in patients with acute myeloid leukemia at day 14 of induction therapy

**DOI:** 10.1186/s13000-015-0365-2

**Published:** 2015-07-25

**Authors:** João Tadeu D Souto Filho, Monique M Loureiro, Wolmar Pulcheri, José Carlos Morais, Marcio Nucci, Rodrigo D Portugal

**Affiliations:** University Hospital, Universidade Federal do Rio de Janeiro, Rua Prof. Rodolpho Paulo Rocco, 255, Sala 4A 12, Rio de Janeiro, 22251-030 RJ Brazil; Faculdade de Medicina de Campos, Campos dos Goytacazes, RJ Brazil; Instituto Federal de Educação, Ciência e Tecnologia Fluminense, Campos dos Goytacazes, RJ Brazil

**Keywords:** Acute myeloid leukemia, Bone marrow, Blasts counting, D14

## Abstract

**Background:**

Early assessment of response to chemotherapy in acute myeloid leukemia may be performed by examining bone marrow aspirate (BMA) or biopsy (BMB); a hypocellular bone marrow sample indicates adequate anti-leukemic activity. We sought to evaluate the quantitative and qualitative assessment of BMA performed on day 14 (D14) of chemotherapy, to verify the inter-observer agreement, to compare the results of BMA and BMB, and to evaluate the impact of D14 blast clearance on the overall survival (OS).

**Methods:**

A total of 107 patients who received standard induction chemotherapy and had bone marrow samples were included. BMA evaluation was performed by two observers using two methods: quantitative assessment and a qualitative (Likert) scale. ROC curves were obtained correlating the BMA quantification of blasts and the qualitative scale, by both observers, with BMB result as gold-standard.

**Results:**

There was a significant agreement between the two observers in both the qualitative and quantitative assessments (K_w_ = 0.737, *p* < 0.001, and r_s_ = 0.798, *p* < 0.001; ICC = 0.836, *p* < 0.001, respectively). The areas under the curve (AUC) were 0.924 and 0.946 for observer 1 and 0.867 and 0.870 for observer 2 for assessments of the percentage of blasts and qualitative scale, respectively. The best cutoff for blast percentage in BMA was 6 % and 7 % for observers 1 and 2, respectively. A similar analysis for the qualitative scale showed the best cutoff as “probably infiltrated”. Patients who attained higher grades of cytoreduction on D14 had better OS.

**Conclusions:**

Evaluation of D14 BMA using both methods had a significant agreement with BMB and between observers, identifying a population of patients with poor outcome.

## Background

The outcome of patients with acute myeloid leukemia (AML) has improved substantially over the past decades, thanks to the development of more aggressive therapies and better supportive care. However, a substantial proportion of patients still do not obtain complete remission (CR), and others eventually relapse after achieving CR [1–3]. In an attempt to stratify subgroups with different survival rates, several prognostic factors have been identified, including age, gender, baseline white blood cell count, lactic dehydrogenase serum level, immunophenotype, karyotypic abnormalities and genetic profiles [4–7].

In addition to baseline variables, early assessment of response to chemotherapy may help to define prognosis. Previous studies have shown an association between the lack of early blasts clearance and failure to obtain CR after a first cycle of induction [8, 9]. This early assessment of treatment response is usually performed between the 14th (D14) and 17th day of the first cycle of induction chemotherapy, by analyzing the cellular content of the bone marrow aspirate (BMA) and/or biopsy (BMB). A hypocellular bone marrow sample suggests adequate anti-leukemic activity [8, 10]. However, its interpretation may be inaccurate because of different levels of expertise among pathologists and hematologists, and a great variability in BMA and BMB sample quality [11]. Furthermore, a BMA blast count above which poor response to chemotherapy is predicted has not been clearly defined, with values ranging from 5 % to 40 % [8–19]. By contrast, the BMB provides a better assessment of marrow cellularity [20], but the results are available only a few days after the BMA, delaying the decision to administer a second course of induction chemotherapy for non-responders.

Given these uncertainties, we sought to evaluate the quantitative and qualitative assessment of D14 BMA, to verify the inter-observer agreement, and to compare the results of BMA and BMB. We also assessed the impact of D14 blast clearance on the overall survival (OS).

## Methods

### Study population and treatment

All patients diagnosed with AML at University Hospital Clementino Fraga Filho, Universidade Federal do Rio de Janeiro (UFRJ) Brazil, from January 1979 to December 2008 were retrospectively evaluated. Entry criteria for this study included: a diagnosis of AML other than acute promyelocytic leukemia, no previous treatment in other institution, receipt of standard induction chemotherapy (cytarabine + antracycline), and performance of BMA on D14 of induction chemotherapy. The study was approved by the local ethics committee (Hospital Clementino Fraga Filho/Universidade Federal do Rio de Janeiro, CAAE n°. 0094.0.197.000-09) and was conducted in accordance with the principles of Helsinki declaration. Informed consent was not obtained due to its retrospective nature of this study did not affect the healthcare of the included individuals. Moreover, confidentiality was preserved.

The diagnosis of AML was based on available procedures at the time, including BMA and BMB, and cytogenetic and immunophenotype analyses. Cases were classified according to de French-American-British (FAB) criteria [21]. The treatment regimens changed over time (Table 1) [22].

**Table 1:** Different approaches to the treatment of acute myeloid leukemia between time periods

Time periods	Treatment
	Induction Remission:
Until 1985	TAD protocol:
	- Thioguanine 100 mg/m^2^ orally every 12 hours for 7 days
	- Cytarabine 100 mg/m^2^/d iv for 7 days
	- Doxorubicin 30 mg/m^2^/d iv for 3 days
After 1985	7 + 3 protocol:
	- Cytarabine 200 mg/m^2^/d iv for 7 days
	- Daunorubicin 45 mg/m^2^/d or doxorubicin 30 mg/m^2^ iv for 3 days
Residual leukemia	5 + 2 protocol:
	- Cytarabine 200 mg/m^2^/d iv for 5 days
	- Daunorubicin 45 mg/m^2^/d or doxorubicin 30 mg/m^2^/d iv for 2 days
	Post-remission treatment:
Until 1985	12 maintenance cycles of TAD
1986 to 1992	4 courses:
	- Cytarabine 400 mg/m^2^/d iv for 3 days
	- Doxorubicin 30 mg/m^2^/d iv for 3 days
1993 to 1998	2-4 courses:
	- High-dose cytarabine 1 g/m^2^ iv every 12 hours for 4 days
	- Doxorubicin 30 mg/m^2^/d iv for 3 days
After 1999	2-4 courses:
	- High-dose cytarabine 3 g/m^2^ iv every 12 hours on days 1, 3 and 5

*iv* intravenous infusion

### Bone marrow aspirate and biopsy

Routine assessments of BMA and BMB were performed on D14 of induction remission. Aspirate smears were prepared at the bedside and stained with Wright-Giemsa stain, and biopsy samples were fixed in 10 % buffered formalin, and stained with hematoxylin and eosin. Patients with persistent disease according to D14 assessment received a second cycle of induction as early as possible [2, 13]. All glass slides were kept in storage units in the hospital achieves.

We reviewed all available slides from BMA performed at diagnosis and on D14. The analysis was performed by two independent observers (board certified hematologists), blinded for patient identification and outcome. The evaluation included confirmation of the initial diagnosis of AML and identification of D14 residual leukemia in a quantitative (percentage) and qualitative (scale) manner. Quantitative evaluation was performed by counting the percentage of blasts in 200 nucleated marrow cells. The qualitative assessment was determined by stratification in a Likert scale [23] of five categories: definitely infiltrated, probably infiltrated, doubtful, probably free and definitely free.

The results of D14 BMB were obtained by reviewing patients’ medical records and registries from the Pathology Service of the hospital. The reports were categorized as aplastic (leukemia free) or infiltrated.

### Statistical analysis

The qualitative assessment of blasts was first treated as an ordinal categorical variable and latter grouped in two categories, and treated as dichotomous categorical variable. Agreement between the two observers was assessed using the kappa coefficient (Cohen’s kappa) and quadratic weighted kappa coefficient (K_w_). The kappa coefficient may range from −1 (complete disagreement) to +1 (complete agreement) and the correlation is usually classified as poor (below 0), mild (0 to 0.2), low (0.21 to 0.4), moderate (from 0.41 to 0.6) substantial (0.61 to 0.8) and almost perfect (0.81 to 1.00) [24]. Further evaluation of the marginal homogeneity of proportions was performed with the McNemar test for dichotomous categorical variables and the McNemar modified test for ordinal categorical variables. In both tests, the presence of a significant *p* value (<0.05) indicates excessive variation between observers [25].

The quantitative assessment of blasts was treated as a discrete variable with a non-normal distribution; comparisons between observers were performed with Spearman’s Correlation Coefficient (r_s_). Measurements between observers were also compared using Intraclass Correlation Coefficient (ICC) and the Bland and Altman method [26].

The D14 BMA evaluation was compared with the BMB (considered as “gold standard”) using receiver operating characteristic (ROC) curves to assess the best cut-off point in terms of sensitivity, specificity and accuracy. The areas under the ROC curves (AUC) were compared using the method of Delong [27]. OS was defined as the time from diagnosis to death of any cause or last follow-up. Survival curves were estimated with the Kaplan-Meier method and differences were compared with the log-rank test. Multivariate analysis for OS was conducted using a Cox model and hazard ratios (HR) were obtained for each observer. All tests were 2-sided, and *p* values <0.05 were considered statistically significant. Statistical analyses were performed using SPSS 11.0 (SPSS Inc., 1989–2001), MedCalc 11.3 and MH Program 1.2142.

## Results

### Patients

Of 295 patients with AML identified in the hospital records, 119 fulfilled entry criteria. Among these 119 patients who had a BMA on D14, we could recover 107 sets of BMA smears, containing samples of the diagnosis and D14 assessment. The median age was 38 years (range 12–77), 12 % were >60 years-old and 58 % were males. In addition, we were able to compare D14 BMA and BMB in 82 patients.

### Agreement analysis between observers

**Fig. 1:**
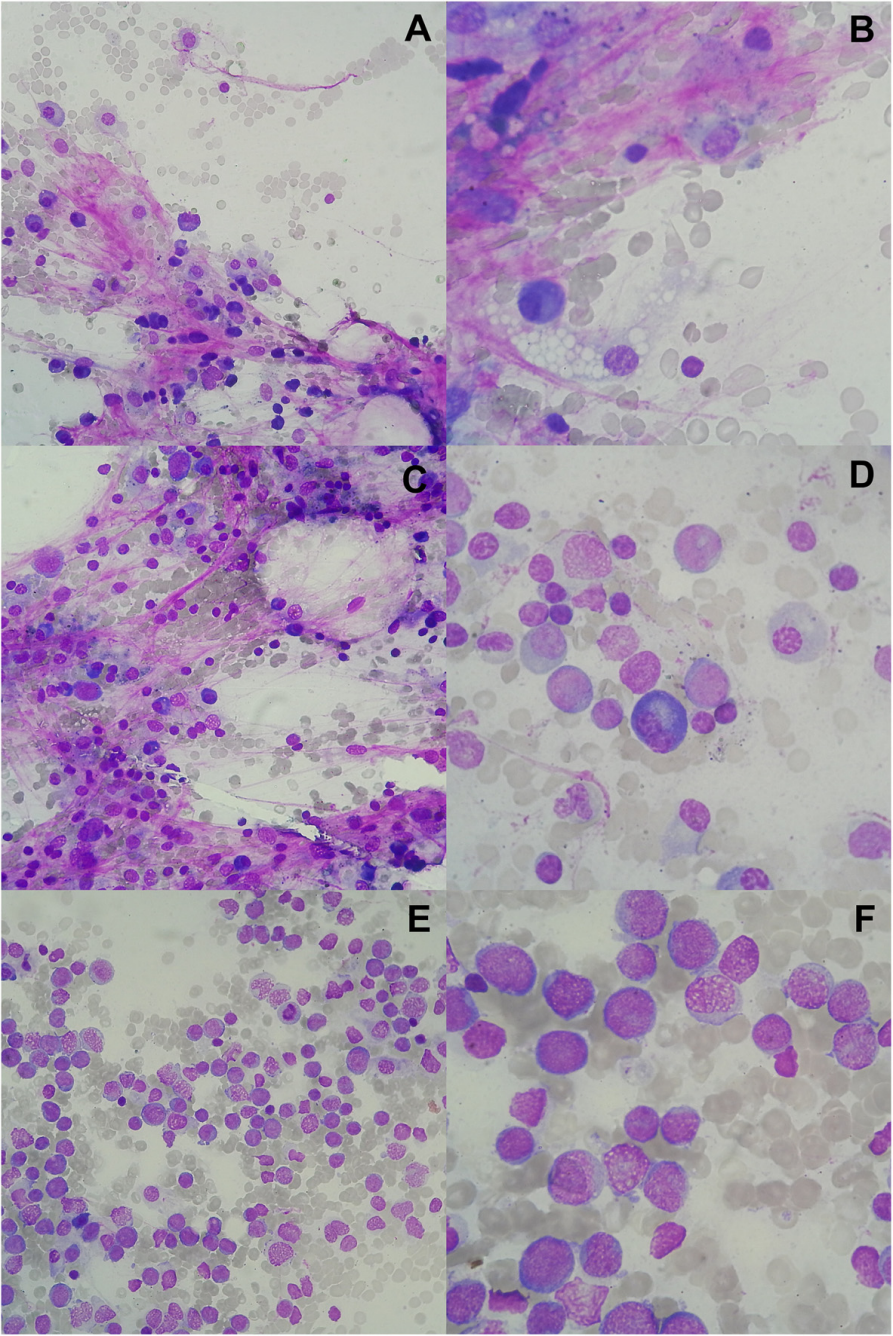
Qualitative assessment of bone marrow aspirates on D14 induction chemotherapy in AML patients. **a** and **b**: definitely free; **c** and **d**: doubtful; **e** and **f**: definitely infiltrated (Wright-Giemsa, x400 and x1000, respectively)

The comparisons between observers of D14 BMA evaluation using the qualitative scale is shown in Table 2. The quadratic weighted kappa coefficient was 0.74 (95 % confidence interval [95 % CI] 0.64 - 0.83, *p* < 0.001), and no bias was observed (*p* = 0.8, modified McNemar test). Typical qualitative categories are shown in Fig. 1.

**Table 2:** Agreement and comparison of frequency between categories of the Likert scale between two observers

Observer 1	Definitely free	Probably free	Doubtful	Probably infiltrated	Definitely infiltrated	Total
Observer 2						
Definitely free	13	4	2	2	0	21
Probably free	17	6	3	7	2	35
Doubtful	0	6	3	1	1	11
Probably infiltrated	1	0	4	9	5	19
Definitely infiltrated	0	0	0	3	18	21
Total	31	16	12	22	26	107

Quadratic weighted kappa coefficient: K_w_ = 0.74, 95 % CI 0.64-0.83, *p* < 0.001)

Modified McNemar test: *X* ^2^ = 0.28, *p* = 0.8

**Fig. 2:**
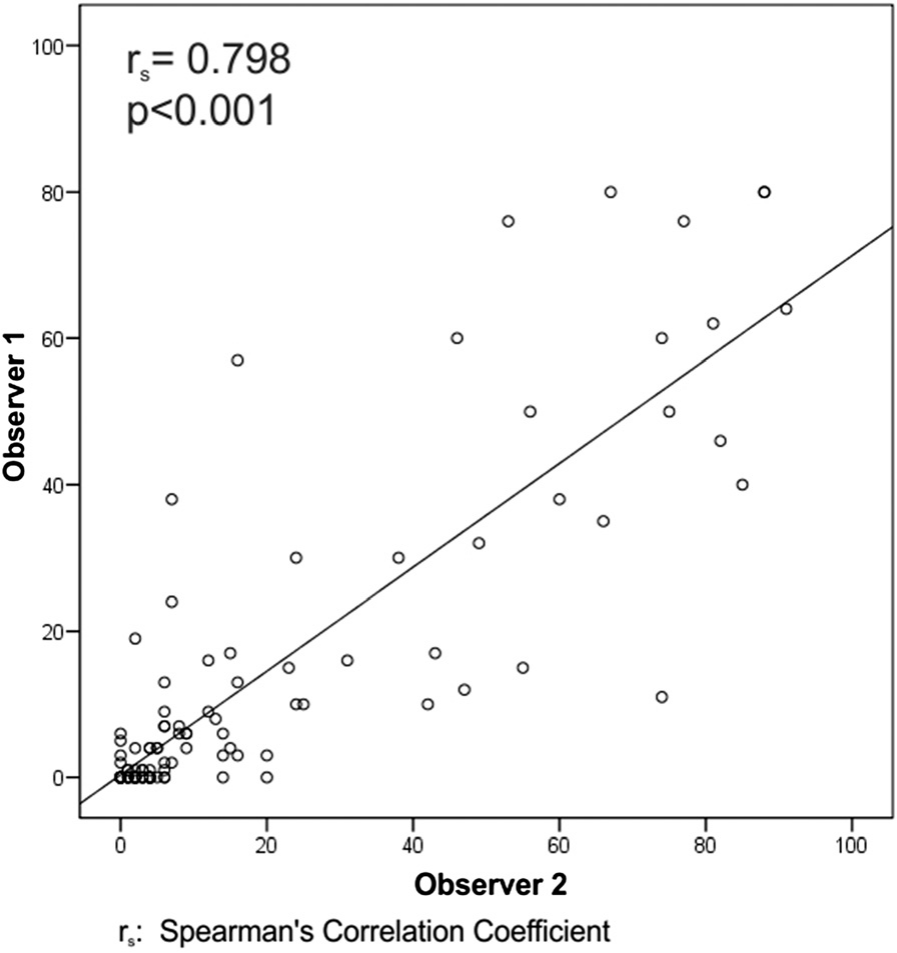
Correlation between the percentage of blasts in D14 bone marrow aspirate by two observers

The median blast count on D14 was 4 % and 6 % for observers 1 and 2, respectively, with a Spearman correlation coefficient of 0.798 (p <0.001) (Fig. 2), and an ICC within assessments of 0.836 (95 % CI 0.768 - 0.885, *p* < 0,001). The average difference between measurements of the percentage of blasts among the observers, according to the Bland and Altman method, was 5.01 % (95 % CI 7.63 - 2.39).

### Comparison of bone marrow aspiration and bone marrow biopsy on D14

The evaluation of BMB on D14 showed 33 patients with bone marrow infiltration and 49 free of leukemia. Table 3 shows the distribution of the categories of the qualitative scale according to the BMB status. We observed an association between the categories of definitely free and probably free with leukemia free in the BMB, and the categories of definitely infiltrate and probably infiltrated with infiltrated BMB (85.4 % for observer 1 and 75.6 % for observer 2). Doubtful results of BMA represented mainly leukemia free BMB for both observers.

**Table 3:** Correlation of BMA evaluation by both observers using the Likert scale with the results of the BMB

	BMB - observer 1		BMB - observer 2	
	*n* = 82		*n* = 82	
BMA	aplasia	infiltrated	aplasia	infiltrated
	*n* (%)	*n* (%)	*n* (%)	*n* (%)
Definitely free	27 (32.9)	0 (0.0)	16 (19.5)	1 (1.2)
Probably free	14 (17.1)	2 (2.4)	23 (28.0)	5 (6.1)
Doubtful	4 (4.9)	2 (2.4)	7 (8.5)	4 (4.9)
Probably infiltrated	3 (3.7)	13 (15.9)	1 (1.2)	9 (11.0)
Definitely infiltrated	1 (1.2)	16 (19.5)	2 (2.4)	14 (17.1)

*BMB* bone marrow biopsy, *BMA* bone marrow aspirate

**Fig. 3:**
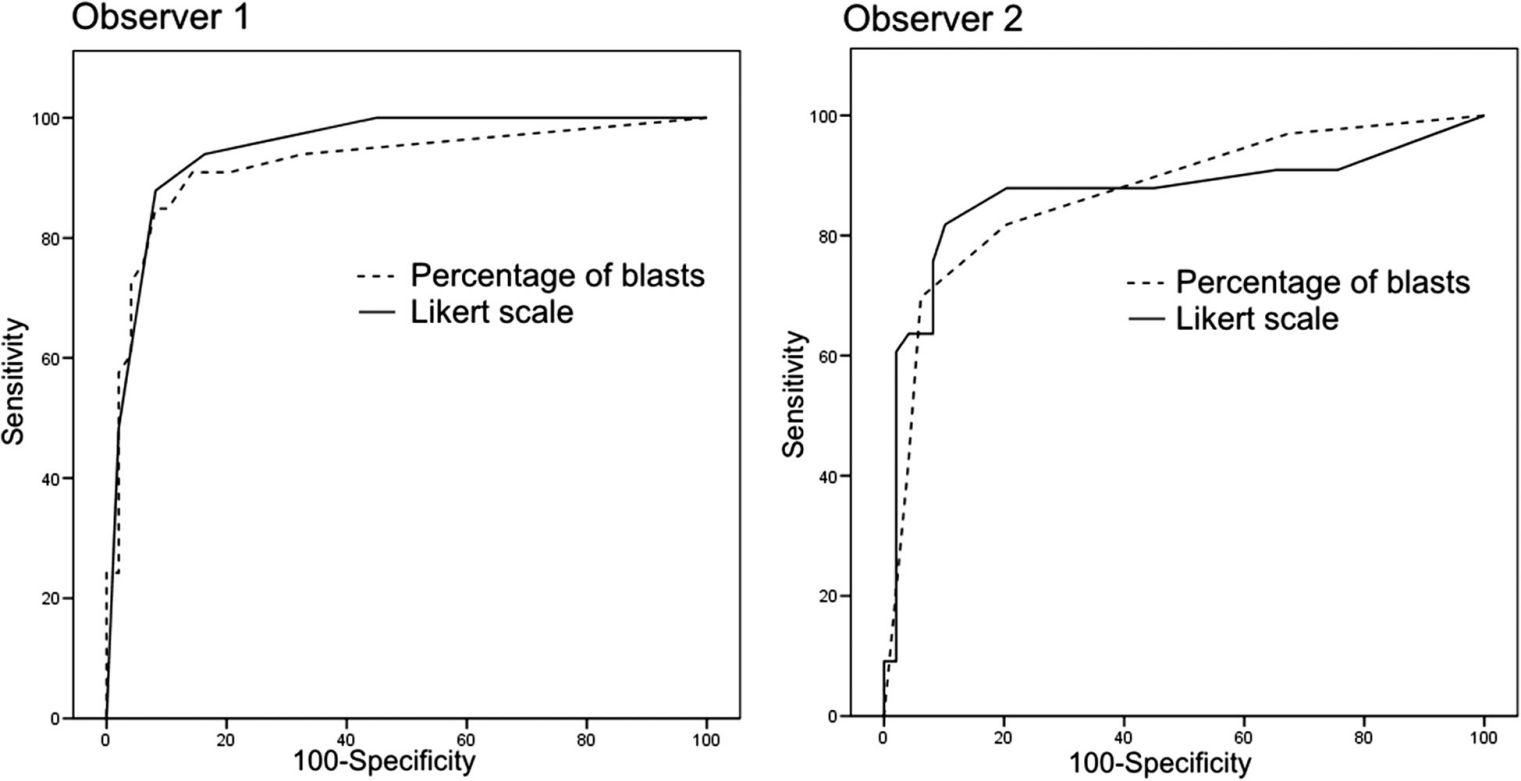
ROC curves of the quantitative and qualitative evaluations of D14 BMA by two observers

Figure 3 shows the ROC curves correlating the BMA quantification of blasts and qualitative scale, by both observers, according to BMB results. The AUCs for the quantitative and qualitative assessments were 0.924 and 0.946 for observer 1, and 0.867 and 0.870 for observer 2, respectively. We also compared the ROC curves of the quantitative and qualitative analysis of each observer. The difference in AUCs was 0.025 for observer 1 (*p* = 0.22) and 0.002 for observer 2 (*p* = 0.97).

### Determining the best cut-off points

The best cut-off points for blast percentage in BMA was 6 % for observer 1 (AUC 0.883, 84.9 % sensitivity, 91.8 % specificity, and 89.9 % accuracy), and 7 % for observer 2 (AUC 0.858, 81.8 % sensitivity, 89.8 % specificity, and 86.6 % accuracy). A similar analysis for the Likert scale showed the best cutoff point as the 4th item of the scale (probably infiltrated) for both observers: AUC 0.898, 87.9 % sensitivity, 91.8 % specificity, and 90.2 % accuracy for observer 1, and AUC 0.818, 69.7 % sensitivity, 93.9 % specificity, and 84.1 % accuracy for observer 2.

Based on the best cut-off point of qualitative assessment, we divided the five categories of the scale in two: “free” and “infiltrated”. The first represents the grouping of categories definitely free, probably free and doubtful, while the second included the categories probably infiltrated and definitely infiltrated. The kappa coefficient for the comparison between observers was 0.66 (95 % CI 0.51 - 0.80, *p* < 0.001), with no bias per McNemar test (*p* = 0.1) (Table 4).

**Table 4:** Agreement and comparison of frequency between grouped categories of the Likert scale between two observers

	Observer 1		
Observer 2	Free*	Infiltrated**	Total
Free*	54	14	68
Infiltrated**	4	35	39
Total	58	49	107

*definitely free, probably free and doubtful; **probably infiltrated and definitely infiltrated

Kappa (K = 0.66, 95 % CI 0.51 - 0.80, *p* < 0.001)

McNemar (*X* ^2^ = 2.28, p = 0.1)

### Impact of D14 blasts on survival

**Fig. 4:**
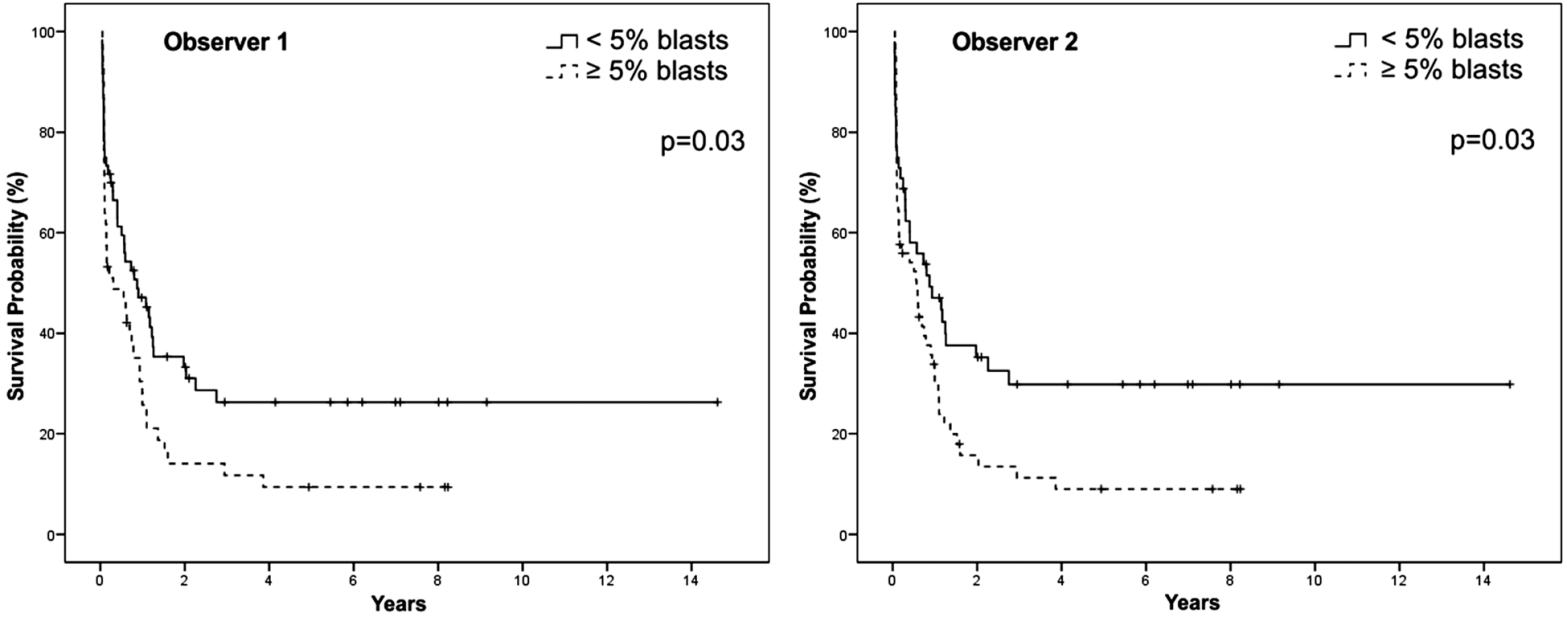
Overall survival according to the quantitative evaluations of D14 BMA by two observers

**Fig. 5:**
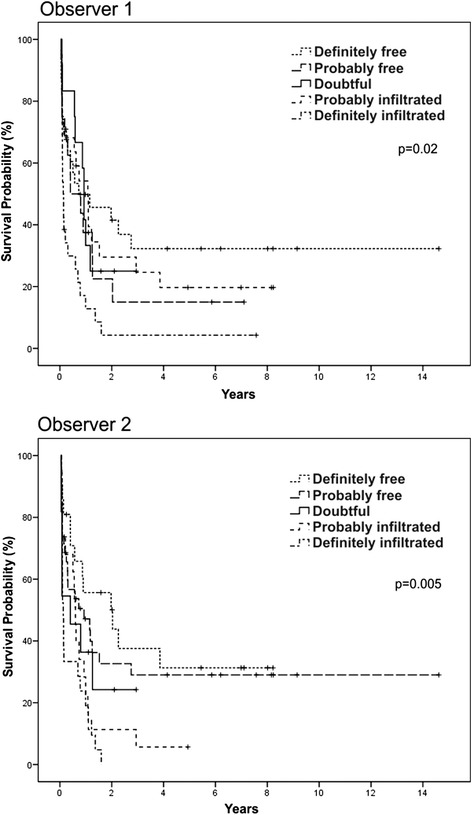
Overall survival according to the qualitative evaluations of D14 BMA by two observers

**Fig. 6:**
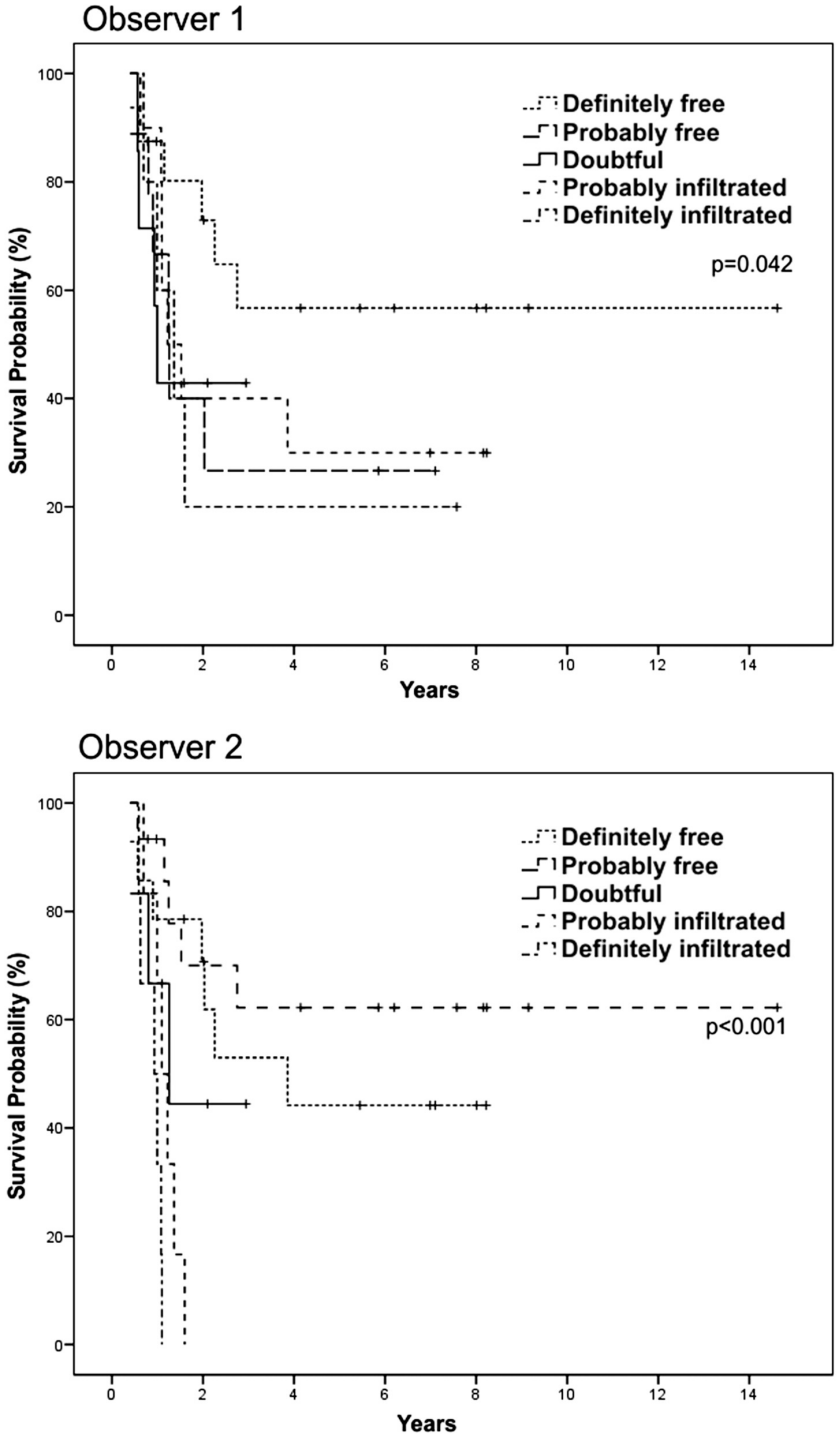
Overall survival according to the qualitative evaluations of D14 BMA by two observers in patients (*n* = 55) treated with two or more cycles of intensification

Five-year OS was significantly longer in patients with <5 % blasts on D14 for both observers (Fig. 4). With Likert scale, a better outcome in patients with lower grades of marrow involvement was also observed (Fig. 5). The same results were obtained among 55 patients in CR who received two or more cycles of intensification (Fig. 6). Other variables detected as prognostic factors by univariate analysis were: age >60 years, year of diagnosis, treatment delay >7 days from diagnosis, presence of comorbidities, previous cardiac disease, hepatomegaly, active bleeding, gastrointestinal infection and FAB subtype M2 (*p* <0.05) (Table 5).

**Table 5:** Factors associated with poor outcome (overall survival) in patients with acute myeloid leukemia by univariate analysis

Variable	HR	95.0 % CI		*P* value
Age > 60 years	1.685	1.203	2.362	0.002
Male gender	1.068	0.799	1.428	0.66
Year of treatment	1.317	1.096	1.582	0.003
Treatment delay > 7 days from diagnosis	1.551	1.031	2.333	0.03
Comorbidities				
Cardiac disease	1.534	1.082	2.174	0.02
Lung disease	1.924	0.937	3.95	0.07
Liver disease	0.880	0.326	2.372	0.80
Kidney disease	2.648	0.976	7.184	0.06
Diabetes	1.371	0.744	2.528	0.31
At least one comorbidity	1.596	1.153	2.211	0.005
MDS-related AML	1.338	0.904	1.979	0.15
Initial clinical manifestations				
Fever	1.265	0.928	1.725	0.14
Gingival hyperplasia	1.268	0.881	1.825	0.20
Lymphadenomegaly	0.867	0.644	1.166	0.34
Hepatomegaly	1.793	1.339	2.400	0.001
Splenomegaly	1.365	0.991	1.88	0.06
Pulmonary involvement	0.668	0.166	2.693	0.57
Cutaneous involvement	0.790	0.404	1.544	0.49
CNS involvement	0.475	0.066	3.401	0.46
Active bleeding	1.589	1.189	2.123	0.002
DIC	0.606	0.225	1.635	0.32
Infection at diagnosis				
Pharyngitis	1.139	0.691	1.876	0.61
Gastrointestinal	2.842	1.439	5.614	0.003
Fever of unknown origin	1.096	0.761	1.578	0.62
Blood	0.632	0.201	1.982	0.43
Skin and soft tissue	0.877	0.575	1.337	0.54
Pneumonia	1.490	0.995	2.23	0.05
Sinusitis	0.392	0.055	2.799	0.35
Oral cavity	0.775	0.462	1.298	0.33
Urinary tract	0.994	0.408	2.421	0.99
At least one infection	1.142	0.843	1.548	0.39
FAB classification				
M0	0.563	0.203	1.560	0.27
M1	0.747	0.367	1.520	0.42
M2	0.524	0.339	0.809	0.004
M4	0.796	0.539	1.177	0.25
M5	1.560	0.963	2.528	0.71
M6	0.888	0.321	2.461	0.82
M7	1.294	0.554	3.025	0.55
Laboratory abnormalities				
Leukocytes	1.000	1.000	1.00	0.13
Neutrophils	0.994	0.985	1.003	0.21
Hemoglobin	1.007	0.981	1.034	0.60
Platelets	1.001	0.999	1.002	0.22
Neutropenia	1.049	0.775	1.419	0.76
Leukocytosis (≥50 × 10^9^/L)	1.220	0.890	1.673	0.22
LDH	1.000	1.000	1.001	0.07
% blasts D14 (observer 1)	1.018	1.009	1.027	0.001
% blasts D14 (observer 2)	1.014	1.007	1.022	0.001
Likert scale D14 (observer 1)	1.197	1.032	1.387	0.02
Likert scale D14 (observer 2)	1.339	1.147	1.563	0.001

*HR* hazard ratio, *95 % CI* 95 % confidence interval, *CNS* central nervous system, *DIC* disseminated intravascular coagulation *FAB* French–American–British, *LDH* lactate dehydrogenase

Predictors of poor outcome (lower OS) by multivariate analysis, with HR obtained respectively for observers 1 and 2, were age >60 years [HR = 4.67 (95 % CI 1.91-11.4) and 4.36 (95 % CI 1.79-10.61)], the presence of active bleeding at diagnosis [HR = 2.37 (95 % CI 1.18-4.74) and 2.05 (95 % CI = 1.01-4.13)] and residual D14 blasts with Likert scale [HR = 1.42 (95 % CI 1.11-1.81) and 1.43 (95 % CI = 1.11-1.92)] (Table 6).

**Table 6:** Factors associated with poor outcome (overall survival) in patients with acute myeloid leukemia by multivariate analysis performed with D14 BMA evaluation by both observers

	Observer 1			Observer 2		
Variable	HR	95.0 % CI	*P*	HR	95.0 % IC	*P*
Age > 60 years	4.676	1.918-11.404	0.001	4.364	1.794 -10.617	0.001
Active bleeding	2.375	1.186 - 4.753	0.01	2.052	1.018 - 4.135	0.04
Likert scale D14	1.425	1.118 -1.816	0.004	1.463	1.114 -1.922	0.006

All variables associated with univariate *P*-value <0.05 were included in the multivariate analysis. Only independent parameters are shown. *HR* hazard ratio; *95 % CI* 95 % confidence interval

## Discussion

In this study we found substantial agreement between observers using two different methods: a quantitative assessment, with the determination of the percentage of bone marrow blasts, and a qualitative, based on the perception of marrow infiltration. In addition, a cutoff value of 6-7 % of blasts in the quantitative assessment and “probably infiltrated” marrow in the qualitative assessment was established, with good discriminatory power to identify patients with infiltrated BMB. Moreover, we observed a higher OS in patients who obtained higher grades of cytoreduction by day 14 marrow evaluation.

While risk assessment in AML relies mainly on age and cytogenetic profile [5], the assessment of in vivo chemosensitivity by determining early response to induction therapy is an additional predictive marker. Indeed, this parameter has been used to guide clinicians in deciding for an early second cycle of chemotherapy [13, 28, 29]. However, the type of D14 bone marrow evaluation (BMA, BMB or both) has varied, with some studies relying on BMA [8, 16], others used BMB [18], and occasionally no clear information was provided [9, 10, 17, 19].

In our study we observed that the qualitative and the quantitative methods were equally predictive of BMB results, with a substantial inter-observer agreement. Bone marrow evaluation by more than one observer has been previously reported [16, 17], but to our best knowledge, our study was the first that reported the assessment of inter-observer agreement.

Another point of controversy is the cutoff values of blast cell percentage in the quantitative assessment of BMA. Different studies have established cutoff values that ranged from 5 % [9, 10, 30, 31], 10 % [8, 9, 17], 15-22 % [16], and even 40 % [10]. These variations are also present in published Guidelines: <5 % [11], <5-10 % [12], <10-15 % [13] and hypoplasia or aplasia (without defining a numerical value) [14]. We established a cutoff value of 6-7 % (inter-observer variation), which is in the range of previous studies, and identified that the qualitative categories of definitely and probably infiltrated were predictive of residual leukemia on BMB.

All analyzes of response assessment by D14 BMA by both methods (qualitative and quantitative) and both observers resulted in higher specificity than sensitivity. Likewise, the concordance between observers was very good for “definitely/probably infiltrated”, but not so good for “definitely/probably free”. Therefore, there is no debate that a large amount of leukemic blast on day 14 constitutes unequivocal evidence of residual leukemia. However, the presence of a few blasts in a paucicellular or hemodilute marrow sample cannot be considered as definite evidence of residual disease. Indeed, most guidelines determine a second induction cycle for unequivocal residual disease and most dilemmas occurs in patients with low blast count (5-15 %) [32].

Few previous studies have shown an association between D14 marrow findings and long-term outcome [8, 9, 10, 17, 30]. In the present study, multivariate analysis showed that the evaluation of the bone marrow infiltration by Likert scale (but not the percentage assessment) was significantly associated with poor outcome.

Our study shares the limitations of all retrospective studies. It was not possible to recover D14 BMA and BMB slides from all cases. In addition, survival analysis was performed without the inclusion of well-known prognostic factors such as chromosomal and molecular abnormalities. Finally, we did not analyze the potential effect of the different induction regimens given throughout the study period and the number of entry-patients over the study period. Despite these limitations, we were able to show that BMA may be considered the procedure of choice to assess treatment response on D14 because it provides results immediately, and exhibited good agreement between observers and good correlation with BMB and OS.

## Conclusions

We conclude that the assessment of BMA on day 14th of remission induction chemotherapy in patients with AML is a reproducible test with a substantial agreement between observers, both quantitatively and qualitatively, has good correlation with BMB and with OS. The percent cut-off 6-7 % or “probably infiltrated” may help to early identify a population of patients with unfavorable prognosis.
